# Multiscale structural gradients enhance the biomechanical functionality of the spider fang

**DOI:** 10.1038/ncomms4894

**Published:** 2014-05-27

**Authors:** Benny Bar-On, Friedrich G. Barth, Peter Fratzl, Yael Politi

**Affiliations:** 1 Department of Biomaterial, Max-Planck-Institute of Colloids and Interfaces, Potsdam 14424, Germany; 2 Department of Neurobiology, Faculty of Life Sciences, University of Vienna, Vienna 1090, Austria

## Abstract

The spider fang is a natural injection needle, hierarchically built from a complex composite material comprising multiscale architectural gradients. Considering its biomechanical function, the spider fang has to sustain significant mechanical loads. Here we apply experiment-based structural modelling of the fang, followed by analytical mechanical description and Finite-Element simulations, the results of which indicate that the naturally evolved fang architecture results in highly adapted effective structural stiffness and damage resilience. The analysis methods and physical insights of this work are potentially important for investigating and understanding the architecture and structural motifs of sharp-edge biological elements such as stingers, teeth, claws and more.

## Abstract (web summary)

Fangs are segments of the spider mouthparts, which are used to inject venom into prey and are required to sustain large mechanical loads. Here, the authors perform experiment-driven simulations, so to understand the correlation between the multiscale structural gradients and the biomechanical function of the fang.

## Graphical abstract

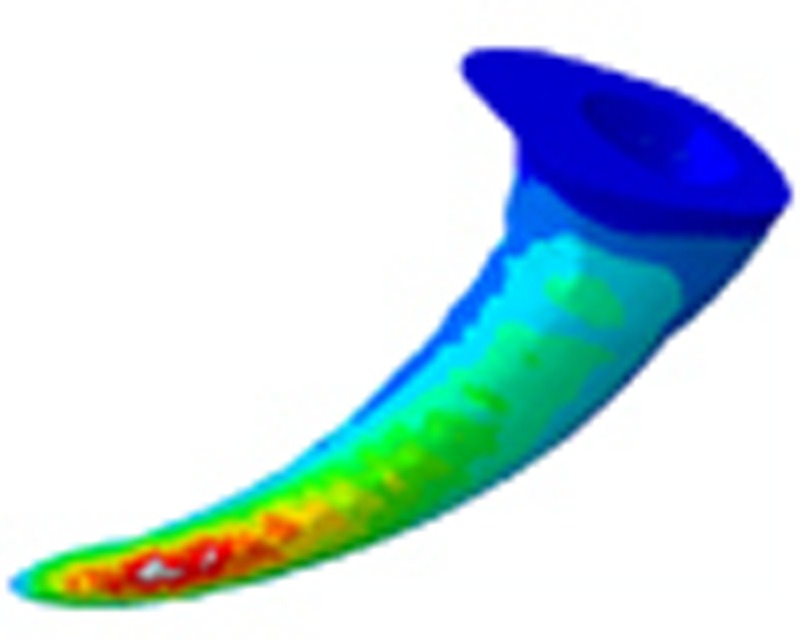

Natural structures adapt their mechanical properties to a large variety of needs, which may be competing, primarily by varying the spatial distributions of their components^1^,^2^. This may result in shape optimization^3^, which is a widely used engineering principle as well^4^. Another kind of adaptation is the use of graded materials, including gradients in composition and microstructural architecture^5^,^6^,^7^,^8^,^9^. Mussel byssal threads, for example, include a distribution gradient of two types of collagenous-proteins. This distribution is directly related to a gradient in the mechanical properties of the thread along its length, with a stiffer distal region and a dissipative proximal region, enabling to resist large impact loads^10^,^11^. Turtle shells include gradients in the architecture of the mineralized collagen fibrils and of the material porosity, forming an efficient light-weight biological armor^12^. Squid beaks exhibit a gradient in the protein cross-linking with a stiff rostrum and compliant base to promote the penetration into a softer prey while protecting the soft buccal tissue^13^. In addition, some cheliceral and jaw elements in invertebrates combine a gradient in the chitin fibrillar architecture with a graded concentration of metal ions that appear to stiffen and harden their cutting edges^14^,^15^,^16^. Understanding the mechanical functionalities of hierarchically graded natural materials is expected to lead to the development of novel bioinspired engineering materials with superior characteristics^9^,^17^,^18^,^19^,^20^. Here we report a study of the spider fang analysing structural gradients at various length scales.

Spiders belong to the arachnid class of arthropods. They mostly feed on insects, another class of arthropods. Their cuticular fangs are part of the spider mouthparts called chelicerae and are connected to their basal segment by a joint and a pair of muscles. Among their several functions such as those in cleaning behaviour and the widening and opening of the egg sac to release the spiderlings, the mechanically most demanding function of the cheliceral fangs is to puncture the cuticle of prey insects in order to inject the venom^21^,^22^,^23^. Spider fangs typically are a few millimetres long, of conical shape, hollow and curved^24^,^25^. The fang is essentially made of two elements, chitin fibres embedded in a protein-rich matrix, which together form a stiff light-weight structure^26^. During an attack, the fangs experience forces at their tips that may result in compression, bending and twisting deformations of the structure. The fang is stiff and its tip is hard enough to penetrate the cuticular exoskeleton of the prey insect, made of chitin and proteins as well, mostly because of the reinforcement of the protein-rich tip by cross-links based on metal ions^25^. Obviously, the fangs are highly resistant to damage that might result from numerous prey captures. Although renewed at each molt, the fangs have to last for more than a year of adult life when no such renewal occurs anymore.

Here we investigate the structural mechanics of the fangs of a large wandering spider (*Cupiennius salei*)^22^. Specifically, we analyse the macroscopic fang stiffness and damage resilience in view of its multiscale architectural motifs using mechanical modelling based on experimental observations from previous work^25^. We first study the macroscopic architecture of the fang and then proceed to the material level. Finally, we discuss the notable characteristics of the natural fang architecture by comparing them with alternative configurations. It is found that both the anatomical shape of the naturally evolved fang and its material-level architecture result in highly adapted effective structural stiffness and damage resilience.

## Results

### A model of the fang structure

**Figure 1:**
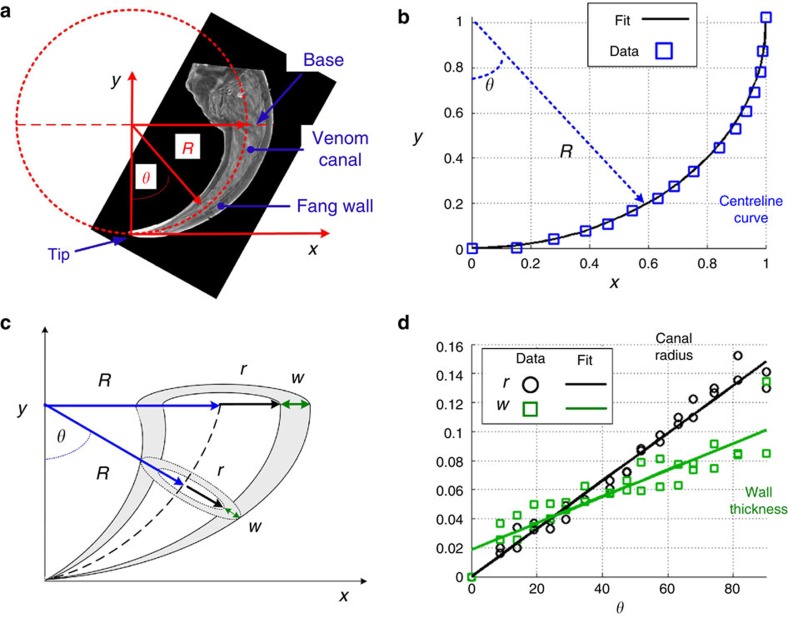
Structure of the natural fang and fang-like model. (**a**) Longitudinal section of the spider fang obtained from X-ray μCT. The red dashed line represents the approximated circular centreline of the fang. (**b**) The fang centreline position as extracted from μCT data. Data points are normalized by the fang radius (*R*=2 mm). (**c**) Structural model of the fang, generated from a centreline with curvature 1/*R*, including a venom canal (radius *r*) and a fang wall of thickness *w*. (**d**) Normalized μCT data for *r*(*θ*) and *w*(*θ*) exhibiting linear trends.

Computer tomography (μCT) reconstruction (Amira 5.1, Visage Imaging GmbH, Germany) was used to analyse the macroscopic architecture of the spider fang. The fang has a curved shape similar to the cat’s claw and includes a venom canal that spans from its base up to close to the tip (Fig. 1a).

The curve that passes through the middle of the venom canal, schematically shown by the dashed line in Fig. 1a, follows the perimeter of a circle of radius *R* (Fig. 1b). On the basis of this insight, we propose an approximated model for the fang structure as shown schematically in Fig. 1c. The model is generated from a centreline with curvature 1*/R* and a running angle 0°≤*θ*≤90°, from which the local axis symmetric cross-section of the fang structure is formed. The local radius of the venom canal *r*(*θ*) and the fang’s local wall thickness *w*(*θ*) are then extracted geometrically from the μCT data. They show a monotonic linear increase from the fang’s tip to its base (Fig. 1d). Thus, the spider fang is viewed here as a hollow conical structure expanding along a quarter-circle curve.

The circular shape of the spider fang indicates that when a moment is applied at the centre of the circle, the tip of the fang is displaced in the circumferential direction, and a penetration force (tangent to the centreline) is generated at the tip. However, the biting mechanism of the spider is more complex^23^,^27^ and the fang may be displaced in non-circular trajectories. As a result, the tangent penetration force at the tip is then accompanied by two auxiliary perpendicular forces in the radial and out-of-plane directions, which result in scratching of the bitten surface. In the following, we investigate how the fang architecture affects the fang’s mechanical properties with regard to these forces. To simplify, a fang-like model with *r*(*θ*)=*w*(*θ*)=*a*_*θ*_(*θ*/90)+*a*_0_ is considered, where *a*_0_ represents the geometry at the tip (*θ*=0) and *a*_*θ*_ represents the steepness of the conical structure—that is, the structural gradient of the fang. Variations in the fang-like model architecture are addressed by variations in the structural gradient (*a*_*θ*_), with *a*_0_ typically being small (*a*_0_/*R*≪1).

### Effect of tapering on the fang’s stiffness

**Figure 2:**
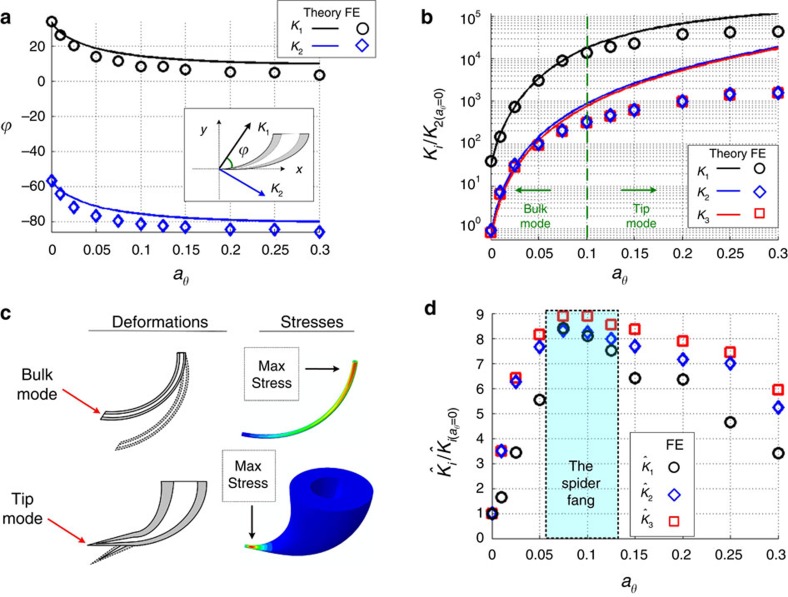
Effects of varying the structural gradient of the fang model on the stiffness parameters. Calculations and FE simulations for 0≤a_θ_≤0.3 and *a*_0_=0.01. (**a**) In-plane orientations of the penetration stiffness (*K*_1_) and perpendicular stiffness (*K*_2_). (**b**) Stiffness parameters *K*_1_, *K*_2_, *K*_3_, normalized by the perpendicular stiffness of a non-graded architecture. Regions of the bulk-dominant and tip-dominant modes are indicated. (**c**) Deformations and stress distributions of the bulk and tip modes. Deformations are schematically illustrated for clarity; stress distributions (von-Mises) are obtained by FE simulations (see Supplementary Information). (**d**) Specific stiffness parameters, 
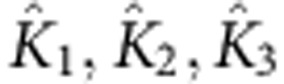
, normalized by the corresponding specific stiffness of the non-graded architecture. The rectangular region indicates the range of the natural fang architecture.

Our stiffness analysis assumed the fang to be fixed at its base, a valid approximation because of the elongated shape of the fang (see Supplementary Note 1). Mechanical forces were applied at the tip (Supplementary Fig. 1). They induce deformation fields along the fang that result in a displacement of the tip from its initial position. The stiffness of the fang is most simply defined in three orthogonal directions, along which the resultant tip displacement is aligned with the force direction, yielding three stiffness parameters (eigenvalues): *K*_1_, *K*_2_ and *K*_3_ (see Supplementary Note 1). *K*_1_ and *K*_2_ are defined in certain directions within the *x*–*y* plane (Fig. 2a) and are in general not aligned with the *x* and *y* axes. *K*_3_ is defined for the out-of-plane direction. The structural gradient of the fang architecture significantly affects these stiffness parameters. This is evaluated here by two approaches: (1) analytical structural mechanics (beam theory)^28^ and (2) numerical Finite-Element (FE) simulations (*Abaqus 6.12*). The analytical approach relies on the assumption that the structural deformations are dominated by the normal and torsional stresses, whereas the effect of shear stresses is assumed to be negligible. These assumptions are usually considered adequate for slender configurations with mild structural gradients. The FE approach, on the other hand, adequately solves the set of differential equations governing the mechanical problem without any simplifying assumptions. Figure 2a,b demonstrates the effect of increasing the structural gradient (that is, conical steepness, *a*_*θ*_) of the fang-like model on the orientation and magnitude of its stiffness. The case of *a*_*θ*_=0 represents a curved needle-like architecture with no structural gradient, and *a*_*θ*_=0.3 represents a steep conical architecture in which the venom canal and the fang wall are much thicker at the base than at the tip. Figure 2a indicates that as the structural gradient increases the *K*_1_ direction becomes closer to being tangent to the tip, and *K*_1_ is therefore identified as the ‘penetrating stiffness’ of the fang. In accordance, *K*_2_ and *K*_3_ are defined as ‘perpendicular stiffness’ parameters. Figure 2b indicates that the penetration stiffness is significantly greater than the perpendicular stiffness parameters (*K*_1_>>*K*_2_≈*K*_3_) and that all stiffness parameters monotonically increase as the structural gradient increases. Interestingly, the needle-like fang architecture (*a*_*θ*_=0) exhibits the lowest penetration stiffness, oriented at ~\n30° to the tip direction. This ‘conventional’ engineering solution is therefore less efficient than are the conical configurations.

Figure 2a,b shows that in case of mild structural gradients (*a*_*θ*_<0.05) the analytical and FE results are compatible, indicating that bending and twisting moments dominate the deformations. In this case, significant stresses and strains are localized close to the base of the fang deforming the entire structure (‘Bulk mode’, Fig. 2c). As the structural gradients increase and the fang architecture becomes more conical, the analytical solution deviates from the FE simulations indicating that shear stresses, which are neglected in the analytical method, become more significant. Specifically, in architectures with steep structural gradients (*a*_*θ*_>0.2) the stresses and deformations are localized more towards the tip, and the rest of the structure is effectively non-stressed and non-deformed (‘Tip mode’, Fig. 2c). The transition between the bulk-dominated and the tip-dominated modes occurs around *a*_*θ*_≈0.1.

An increase in the structural gradient increases the stiffness of the fang but also the volume of the structure and its material content. We therefore look at the specific stiffness parameters (stiffness-per-volume), 
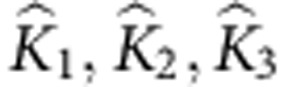
. Figure 2d shows that the specific stiffness parameters are maximized for a range of structural gradients in the vicinity of *a*_*θ*_≈0.1. This region, previously identified as the bulk-to-tip mode transition, represents the fang architectures optimal with regard to structural gradients and specific structural stiffness.

Considering the above results for the fang-like model, we now proceed to analyse the natural fang architecture. The natural configuration is more complex than the fang-like model because of different structural gradients in its venom canal (*a*_*θ*_=0.14) and wall thickness (*a*_*θ*_=0.06), and an imperfect axis symmetric cross-sectional architecture (Fig. 1). To evaluate the stiffness of the natural configuration, FE simulations were performed with the natural 3D fang structure reconstructed using X-ray μCT. The resultant stiffness parameters of the natural fang were then compared with the results received for the fang-like model and found to fit the range of structural gradients of 0.075<*a*_*θ*_<0.1. Thus, the architecture and stiffness of the natural fang configuration are comparable to the architecture parameters of the fang-like model (Fig. 2d) found to be optimal by FE analysis.

### Stress distributions

**Figure 3:**
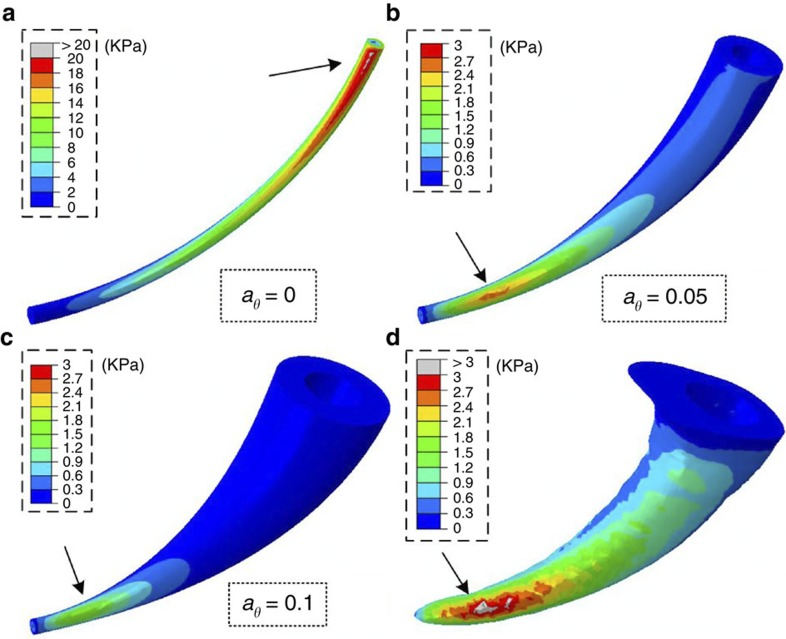
FE simulation. Von-Mises stress (KPa) distributions in the fang model with different structural gradients. (**a**) *a*_*θ*_=0, (**b**) 0.05, (**c**) 0.1 and *a*_0_=0.01, and (**d**) in the natural configuration based on μCT reconstruction. Isotropic loading conditions are considered in all three orthogonal directions (1 N in each). The arrows in the figure indicate the location of the maximal stress. Note the different scale bar in **a**.

In addition to the stiffness variations, the structural gradients in the fang architecture affect the stress distribution and therefore strength and resilience. To evaluate these effects, von-Mises stress distribution maps (Fig. 3a–c) were calculated by FE simulations for the fang-like model with selected architectures, *a*_*θ*_=0, 0.05, 0.1 and *a*_0_=0.01, for the general case of isotropic loading in all three orthogonal directions. The results indicate that the maximal stresses decrease as the structural gradient (*a*_*θ*_) increases and that the maximal stress for the non-graded needle-like architecture (*a*_*θ*_=0) is within an order of magnitude higher than for the conical architectures. Moreover, the stress maximum is shifted from close to the base for the needle-like structure towards the tip for the conical structure.

The stress distribution in the natural fang is shown in Fig. 3d. It is similar to that determined for the fang-like model for *a*_*θ*_≈0.05. Note, however, that the stress intensity in the natural configuration is higher than in the fang-like model, possibly because of the thinner walls and wider canal of the natural fang, which were not considered in the model.

### Architecture at the material level

**Figure 4:**
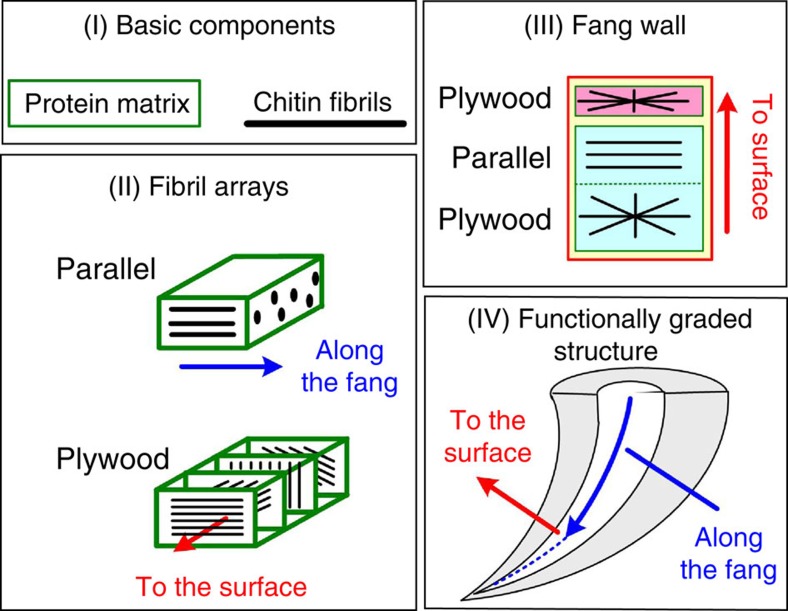
Schematic description of the hierarchical spider fang structure. (level I) the basic components, (level II) fibril arrays, (level III) layered fang wall and (level IV) whole fang.

So far, we focused on the macroscopic structural gradients of the spider fang and considered the material to be homogeneous and isotropic. However, as the rest of the spider’s cuticular exoskeleton, the fang is composed of a composite material of stiff chitin nanofibrils and a more compliant proteinous matrix^29^. The chitin–protein components are assembled into larger-scale architectures, which eventually form the macroscopic fang structure (Fig. 4I–IV)^25^. We consider two levels of material organization: (level II) fibre arrangement within the matrix and (level III) variations in the superposition of these arrangements in the fang wall. The basic composite unit (level II) includes two types of fibril arrays: parallel-fibred and rotated-plywood (Fig. 4II). The parallel-fibred array is composed of fibrils that are locally aligned with the longitudinal axis of the fang (Fig. 4IV). The rotated-plywood array is composed of chitin–protein sheets stacked parallel to the fang surface, where each sheet includes a single layer of unidirectionally oriented fibrils. The fibril orientation varies between successive layers, resulting in a lamellar structure for each 180° rotation of the fibrils. On the next structural level (III), the fang wall is composed of layers of rotated-plywood and parallel-fibred arrays (Fig. 4III). The wall architecture can be divided into two regions: (1) thin rotated-plywood surface layer, corresponding to the so-called exocuticle and (2) a thick bulk region composed of a parallel-fibred array laid over an innermost rotated-plywood layer (the meso- and endocuticle)^23^,^25^. The thickness of the surface layer is effectively uniform along the entire fang, whereas the thickness of the bulk region gradually increases from tip-to-base, resulting in the macroscopic conical shape (Fig. 4IV). In the bulk region, variations in the relative thickness of parallel-fibred and rotated-plywood layers have been reported^25^.

### Chitin fibre architecture and stiffness

Analytical mechanics of composite material methods and a bottom-up modelling approach^30^,^31^ are applied to estimate the effect of the fang composite architecture on the structural stiffness on the two levels described above. The elastic moduli of the fibril arrays (level II) are examined as a function of their constituents and are then used to examine the effect of different layer organizations (level III) on the fang overall stiffness.

**Figure 5:**
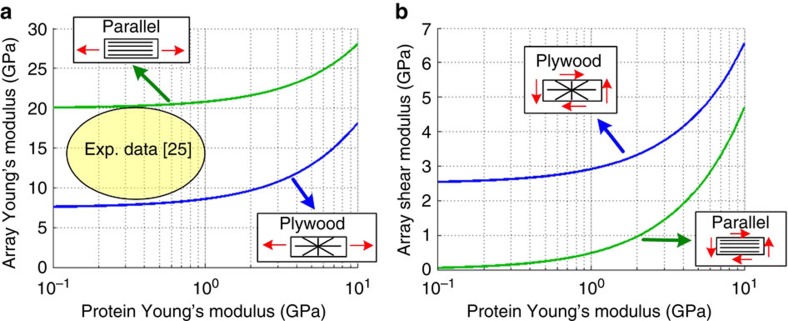
Effect of the proteins’ Young’s modulus (level I) on the composite moduli (level II). (**a**) Young’s modulus and (**b**) shear modulus of parallel-fibred and rotated-plywood fibril arrays, made from chitin fibrils (modulus ~\n100 GPa, volume fraction 0.2) and a protein matrix (range of modulus). The circle in **a** indicates the typical range of the experimental values^25^.

The chitin–protein architecture has a significant effect on the elastic properties of the layers composing the fang wall. This is demonstrated in Fig. 5 for a range of protein elastic moduli (~\n0.1–10 GPa), for a typical modulus of the chitin fibrils (~\n100 GPa) and a typical chitin volume fraction (~\n20%)^29^. On the basis of the experimental measurement of Young’s modulus by applying nanoindentation on the fang composite material^25^ and on the theoretical curves in Fig. 5a, the modulus of the fang proteins is estimated to assume values between ~\n0.1 and 1 GPa (see Fig. 5a). The parallel-fibred layer in the fang wall provides high axial stiffness that resists the applied axial forces and bending moments. However, it provides only poor shear stiffness and can hardly resist the twisting moments (Fig. 5a). The plywood layers, on the other hand, provide a high shear stiffness. Shear stiffness is between five times and two orders of magnitude larger than in the parallel-fibred array, which resists the twisting moments (Fig. 5b). More details on the stiffness of these fibril arrays are provided in Supplementary Note 2. Note that in case of the biologically relevant fibril fraction, the out-of-plane modulus (perpendicular to the layers) is in the order of magnitude of the protein modulus and has only a minor effect on the structural stiffness.

**Figure 6:**
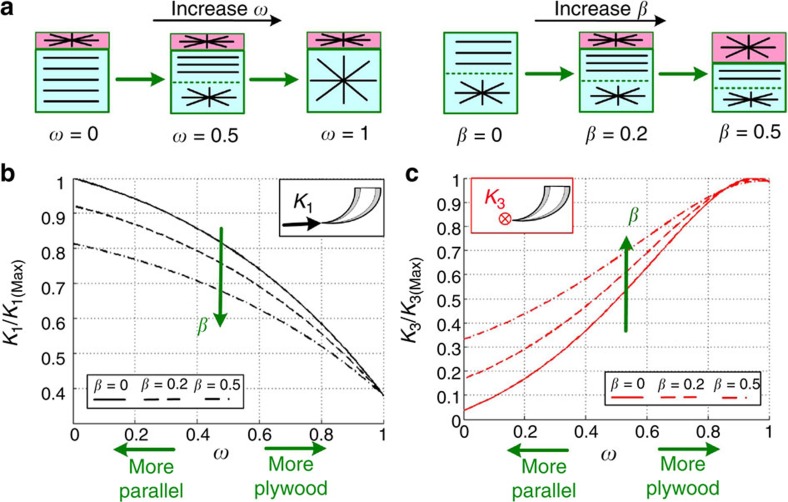
Effect of the layered composite architecture (level III) on the fang’s macroscopic stiffness (level IV). (**a**) Fang wall structure as a function of increasing *β* and *ω*. (**b**,**c**) Effect of variations in *β* and *ω* on the penetration stiffness (*K*_1_) and the perpendicular stiffness (*K*_3_). The results are normalized by the maximal stiffness values.

At the next level considered (Fig. 4, level III) we define two parameters, *β* and *ω*, to describe the layered fang wall architecture (Fig. 6a): *β* is the surface layer thickness relative to the wall thickness at the tip, where in the natural fang structure *β*≈0.2 (ref. 25), and *ω* is the relative portion of the inner plywood layer in the bulk region. Thus, *ω*=0 represents a purely parallel-fibred array and *ω*=0.5 an equal thickness of the parallel-fibred and plywood layers. The penetration stiffness *K*_1_ and the perpendicular in-plane stiffness *K*_2_ of the composite fang are both dominated by the axial stiffness of the parallel-fibred array, while the perpendicular out-of-plane stiffness *K*_3_ is dominated by the shear stiffness of the plywood layers. Figure 6b,c demonstrates the effects of variations in the fang wall architecture (*β*, *ω*) on the *K*_1_ and *K*_3_ stiffness parameters. The results for *K*_2_ are almost identical to those for *K*_1_ and therefore not shown. The penetration stiffness of the fang increases as the parallel-fibred portion in the fang wall increases (that is, smaller *β*, *ω*), whereas the perpendicular stiffness increases as the plywood portion increases (that is, higher *β*, *ω*; Fig. 6). A fully parallel-fibred architecture (*β*→0, *ω*→0) provides the highest penetration stiffness but the lowest perpendicular stiffness. A fully plywood fang architecture (*ω*→1) exhibits the opposite features. Figure 6 also indicates that the thin surface layer of the fang has a significant effect on the structural stiffness, which is especially pronounced for a fang architecture dominated by parallel fibres (*ω*→0). Note that at the biologically relevant configurations (especially the low fibril contents) considered, variations in the properties of the fang material such as the fibril-to-matrix stiffness ratio have only mild effects on the stiffness parameters of the fang structure (Supplementary Fig. 3).

### Gradients in material properties

**Figure 7:**
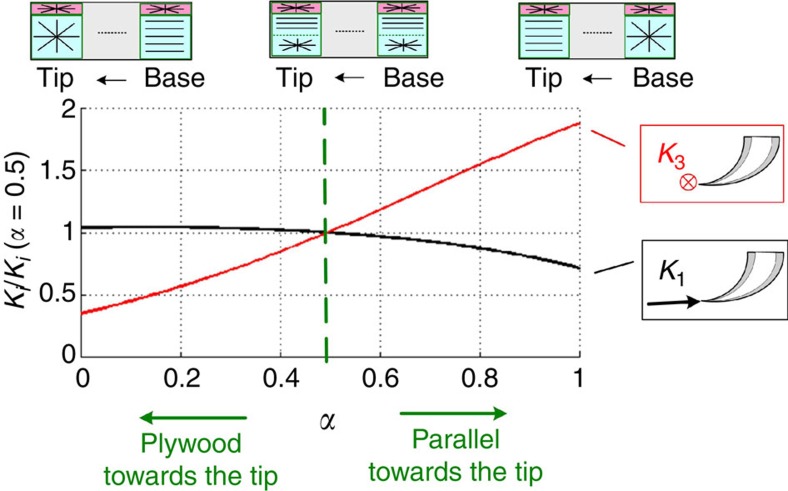
Effect of linear gradients within the composite architecture on *K*_1_ and *K*_3_. (0≤*α*≤1, see Supplementary Fig. 4). The results are normalized by the stiffness of the non-graded architecture (*α*=0.5).

Experimental observations indicate that the elastic modulus and hardness of the fang material are significantly enhanced close to the fang tip, and that the degree of fibril alignment along the fang axis decreases from the tip to the base of the fang^25^. The former effects are attributed to local variations in the matrix proteins and to the incorporation of metal ions, which act as cross-linkers of the matrix. The latter effect is attributed to gradients in the parallel-plywood portions of the fang wall. The increase in the tip modulus only negligibly affects the structural stiffness, but the tip’s increased hardness might enhance the damage resilience against the tip-intensified stress fields (Fig. 2). The effect of gradients in fibril orientation on the stiffness is studied by considering a linear variation in the parallel-to-plywood ratio along the fang (Supplementary Fig. 4). This ratio varies between *ω*(*θ*=0)=*α* at the tip and *ω*(*θ*=90)=1−*α* at the base of the fang (0≤*α*≤1). Whereas *α*=0 represents a fang with plywood architecture at the tip and a parallel-fibred architecture at the base, *α*=0.5 represents a non-graded configuration of equally thick plywood and parallel-fibred layers all along the fang (*ω*=0.5). The gradients in the fibril orientation only mildly affect the penetration stiffness of the fang but strongly affect its perpendicular stiffness (Fig. 7). The natural fang architecture is dominated by plywood arrangement at the base and by a parallel-fibre arrangement at the tip (*α*→1)^25^. This graded architecture goes along with slightly less penetration stiffness and much larger perpendicular stiffness than in case of a uniform configuration (*α*=0.5).

## Discussion

A spider’s fang has to safely sustain high mechanical loads. Although most of its structural features are well documented^25^, their contributions to its overall mechanical properties were yet to be identified. According to our results, the fang architecture is well adapted with a close-to-optimal mechanical stiffness, as defined by the simplified fang-like model and refined damage resilience. A number of possible complications, however, were not considered in the present analysis—for example, the potential variability of the protein matrix and the role of the muscles moving the fang.

On the macroscopic scale, the curved fang structure enables the spider to attack from different directions and to hold its prey like a claw^3^,^23^,^32^,^33^,^34^. This could hardly be achieved by non-curved injection devices—for example, a mosquito stylet or a bee stinger^35^. The hollow conical fang architecture provides the highest structural stiffness available for minimal material content and brings the ‘penetration stiffness’ in a direction almost tangent to the tip. The tapering also enhances the load bearing of the structure (10 times greater than the needle-like structure) and yields a steep reduction in the stress magnitude from the fang’s tip to its base (functioning similar to a bumper). In extreme loading conditions, localized damage is expected only in the near-tip region, after which the fang could still be used. The needle-like (non-graded) configuration is significantly less efficient than the conical architecture. It is considerably more compliant, with its penetration stiffness at an angle to the tip, and at risk of catastrophic damage (full fang truncation) because of high stress concentrations near the base. In experiments where spiders were stimulated to bite into a strain gauge device instead of a real insect prey, the highest value for the force exerted by the fangs measured ca.1N (0.84±0.1 N). In these experiments, no damage to the fangs was ever observed^23^.

On the material level, the fang is a chitin–protein composite, combining both unidirectional (parallel-fibred) and quasi-isotropic (rotated-plywood) architectures. While non-curved biological structures typically are required for a high axial stiffness and usually include unidirectional fibre arrangements only^31^,^36^, the fang is subjected to more complex loading conditions. It therefore includes a graded architecture combining parallel-fibred and rotated-plywood arrangements. The parallel-fibred part provides stiffness resisting axial forces and bending moments, whereas the plywood regions provide shear stiffness resisting twisting moments. The rotated-plywood architecture is a generic structural motif in many organisms^37^. Possibly other architectures^26^ have evolved from the rotated plywood to support more specific mechanical requirements.

The multi-scale structural gradients discussed here are complementary regarding the fang’s mechanical functionality, which requires both high penetration stiffness and moderate perpendicular stiffness. The macroscopic structural gradients increase the fang’s specific stiffness in all directions; however, the perpendicular stiffness is much lower than the penetration stiffness. The base-to-tip gradient in the plywood-to-parallel composite architecture of the fang significantly enhances the perpendicular stiffness. This effect is further amplified by the thin plywood surface layer coating the fang material. Matrix hardening of the composite material near the tip^25^ protects the structure against the stress fields intensified in this area, thereby enhancing the damage resilience of the fang. Whereas this work focuses on the multiscale fang structure, spanning from the basic continuum material level to the macroscale, future studies at the molecular level of the fang material may provide an even deeper understanding of its hierarchical structure and functional adaptation and optimization^38^,^39^,^40^.

In conclusion, it appears that the multiscale graded structural architecture of the spider fang is well adapted to support its biomechanical functionalities. The present analysis emphasizes the importance of considering different structural scales in an effort to understand the overall mechanical properties of biological structures. Revealing the mechanical significance of the spider fang architecture may contribute to the understanding of the selective pressures that have driven the evolution of a variety of sharp-edge biological structures, ranging from scorpion stinger to mammoth tusk. The present work may also promote innovative designs of bioinspired injection devices for engineering (for example, fueling and cooling) and biomedical (for example, infusion, suction and bypassing) applications.

## Author contributions

B.B. and Y.P. conceived the project. B.B.-O. designed the research, performed the analysis and wrote the manuscript. Y.P., F.G.B. and P.F. co-wrote the manuscript. All authors discussed the results and commented on the manuscript.

## Additional information

**How to cite this article:** Bar-On, B. *et al.* Multiscale structural gradients enhance the biomechanical functionality of the spider fang. *Nat. Commun.* 5:3894 doi: 10.1038/ncomms4894 (2014).

## Supplementary Material

Supplementary Information Supplementary Figures 1-4, Supplementary Notes 1-2 and Supplementary References
