# Supplementary material for: Multiscale structural gradients enhance the biomechanical functionality of the spider fang
Source: Nat Commun. 2014 May 27;5:3894. doi: 10.1038/ncomms4894 (PMC4050259; doi:10.1038/ncomms4894)
Supplement: Supplementary Information — Supplementary Figures 1-4, Supplementary Notes 1-2 and Supplementary References [file ncomms4894-s1.pdf]

Supplementary figures

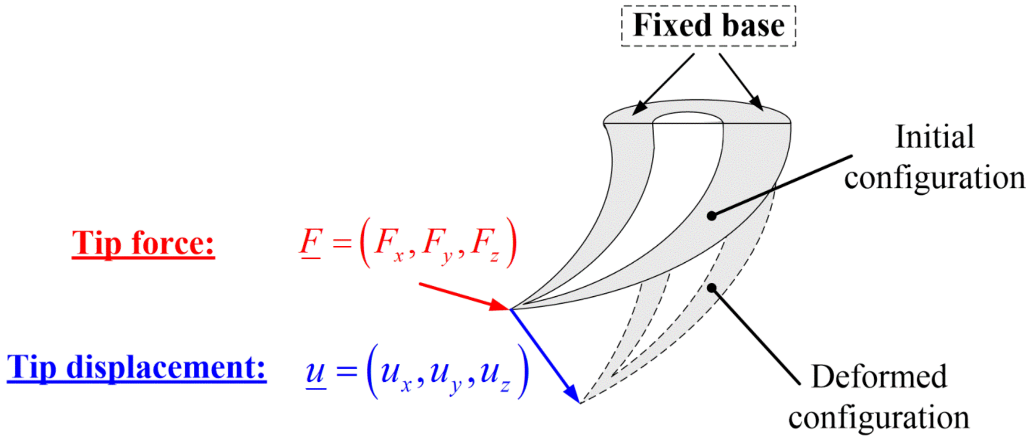

Supplementary figure 1: The fang model (longitudinal section) used for the stiffness analysis. The base of the fang is considered fixed (i.e. zero displacement constraint) and the tip of the fang is subjected to a generic force vector $\underline{F} = (F_x, F_y, F_z)$. As a result, the fang is deformed relative to its initial configuration and its tip displaced by $\underline{u} = (u_x, u_y, u_z)$.

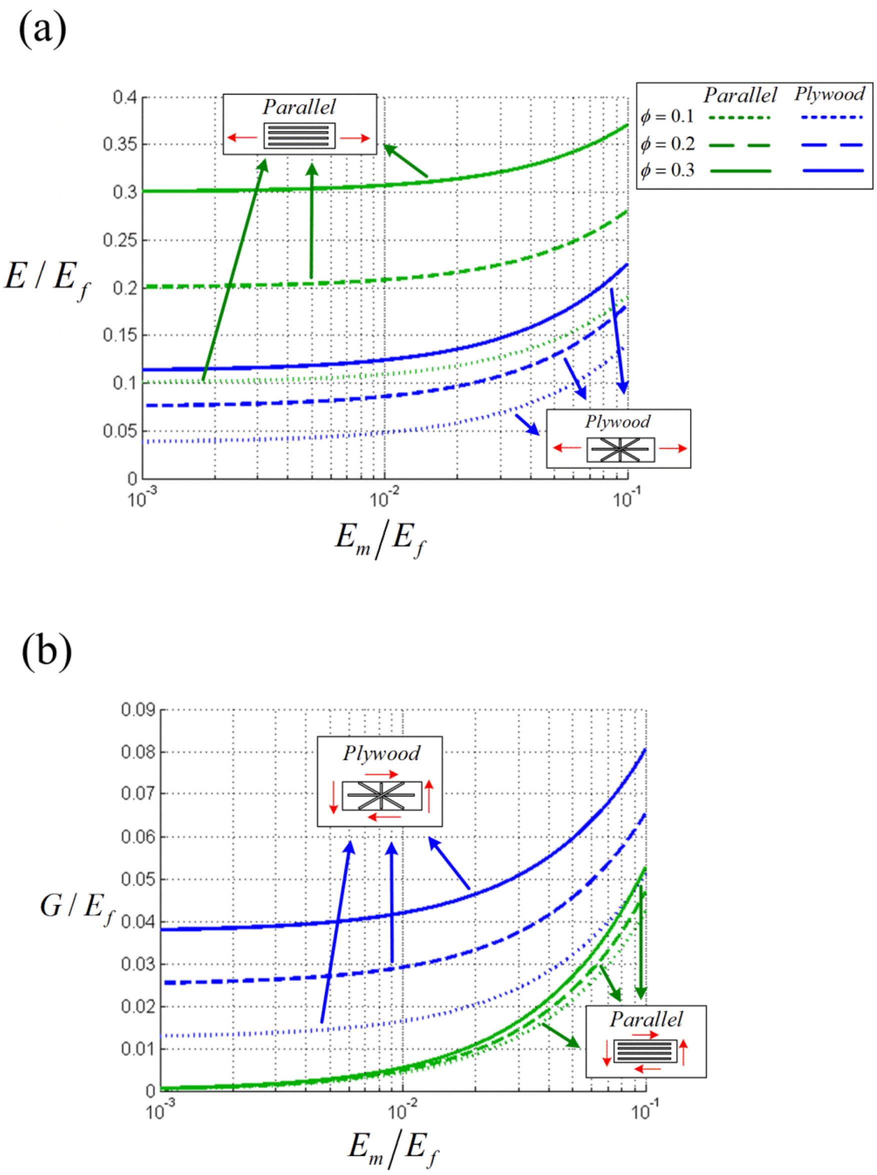

Supplementary figure2: Young's modulus (a) and shear modulus (b) of arrays with parallel fibers and rotated-plywood fibers normalized by E_f, calculated using eqs. (3-6) in supplementary note 2 for a range of matrix-to-fibril modulus ratios ($10^{-3} \leq E_m/E_f \leq 10^{-1}$) ,Poisson ratio $\nu_m = \nu_f = 0.3$, and selected fibril volume fractions $\phi_f = 0.1,0.2,0.3$. The asymptotic relations in eqs. (9-12) in supplementary note 2 are obtained for $E_m/E_f \to 10^{-3}$.

(a)

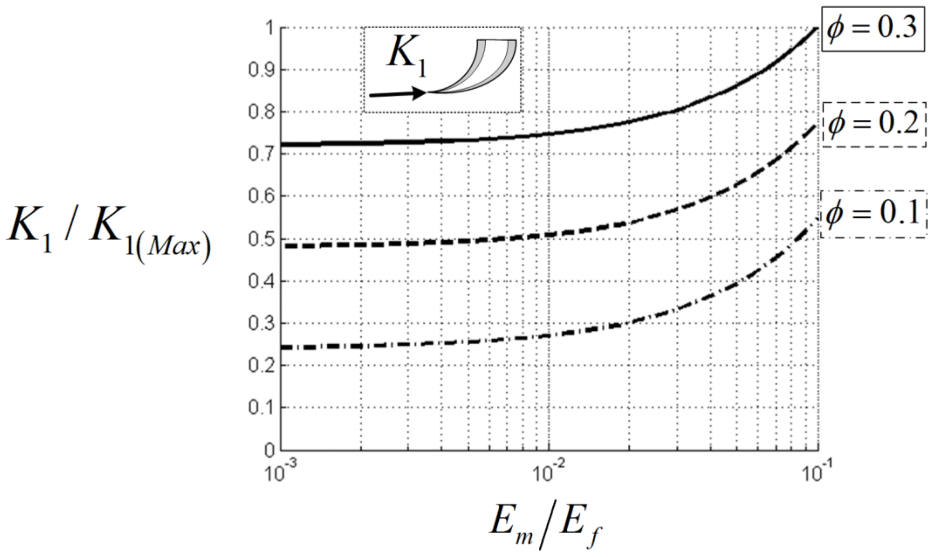

(b)

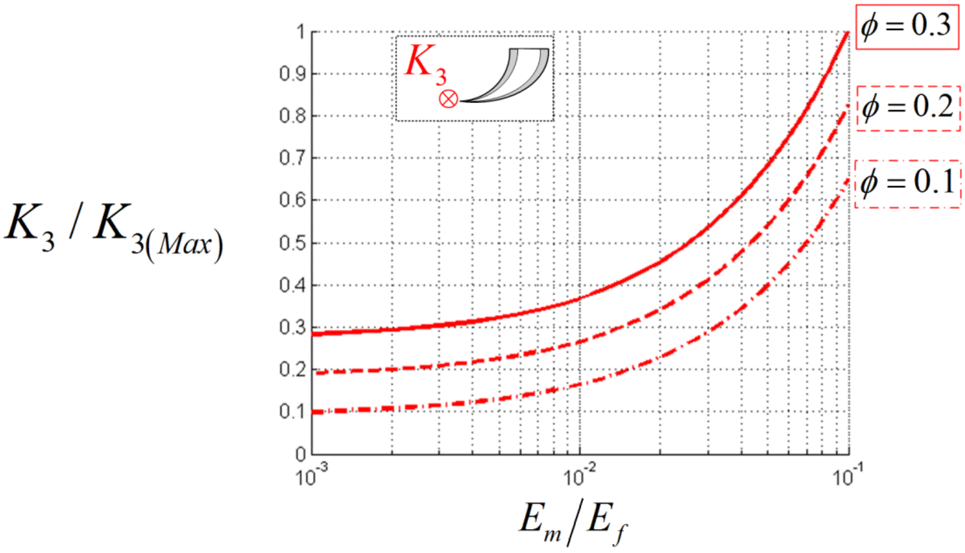

Supplementary figure 3: The penetration stiffness (K_1) (a) and perpendicular stiffness (K_3) (b) of the fang model normalized by their maximal values, calculated for a fang-like architecture of $a_\theta = 0.1$, $a_0 = 0.01$,a range of matrix-to-fibril modulus ratios $10^{-3} \leq E_m/E_f \leq 10^{-1}$, Poisson ratio $\nu_m = \nu_f = 0.3$, and selected fibril volume fractions $\phi_f = 0.1, 0.2, 0.3$.

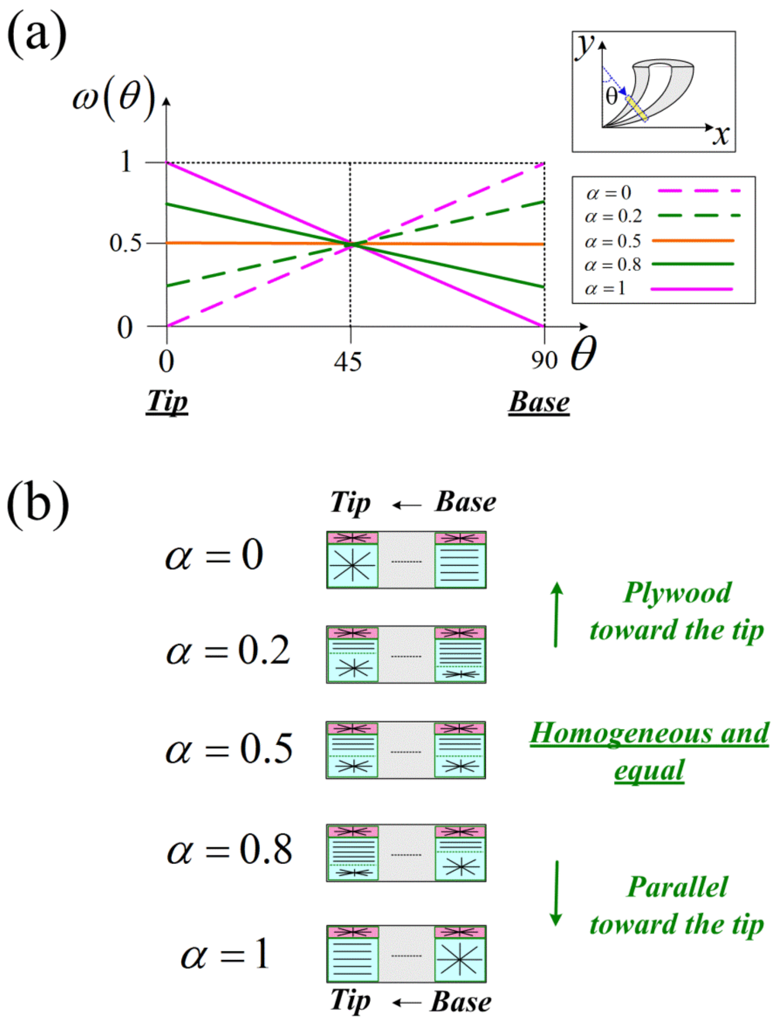

Supplementary figure 4:(a) Linear gradients, in the plywood-parallel portions along the fang, $\omega(\theta)$, are defined in the following way: $0 \le \alpha \le 1$ determines the ω ratio at the tip and at the base via $\omega(\theta = 0) = \alpha$ and $\omega(\theta = 90) = 1 - \alpha$, respectively. The architecture in any position along the fang is obtained by linear interpolation between $\omega(\theta = 0)$ and $\omega(\theta = 90)$. (b) Schematic description of the bulk region architectures at the tip and base of the fang for selected α values. $\alpha < 0.5$ and $\alpha > 0.5$ indicate architectures dominated by rotated-plywood and parallel-fiber dominated arrangements, respectively. For example, for $\alpha = 0.2$ the bulk region at the tip of the fang is composed of 20% rotated-plywood and 80% parallel-fibers. In this case, the base of the fang is composed of 80% rotated-plywood and 20% parallel-fiber array.

Supplementary note 1. Stiffness parameters of the fang structural model

The spider fang´s stiffness was analyzed by considering the fang fixed at its base and subjected to forces at the tip, $\underline{F} = \left(F_x, F_y, F_z\right)$.The fang structure is deformed and its tip displaced, $\underline{u} = \left(u_x, u_y, u_z\right)$ from its unloaded position (see supplementary figure 1). Following the linear elastic theory, $\underline{F}$ and $\underline{u}$ are related via a stiffness matrix as follows:

$$\begin{pmatrix} F_x \\ F_y \\ F_z \end{pmatrix} = \begin{pmatrix} K_{xx} & K_{xy} & K_{xz} \\ K_{xy} & K_{yy} & K_{yz} \\ K_{xz} & K_{yz} & K_{zz} \end{pmatrix} \begin{pmatrix} u_x \\ u_y \\ u_z \end{pmatrix} \tag{1}$$

The elastic energy of the fang structure (U) is a quadratic form of the displacement components and takes both linear bending and torsion effects into account (1).The components of the stiffness matrix are extracted from the second derivative of U with respect to the corresponding displacement components, as follows:

$$K_{ij} = \frac{\partial U}{\partial u_i \partial u_j} \quad ; \quad i, j = x, y, z \tag{2}$$

Due to the symmetry of the structural model there is no coupling between the in-plane force components (F_x, F_y) and the out-of plane displacement (u_z), i.e. $K_{xz} = K_{yz} = 0$.

The stiffness matrix in eq. (1) is now diagonalized to extract its eigenvalues (K_1, K_2 and K_3) and eigenvectors. Two eigenvectors, which correspond to the K_1 and K_2 , eigenvalues are obtained in a certain orientation in the $x - y$ plane (the φ angle in figure 2a); the third eigenvector, which corresponds to K_3,coincides with the out-of-plane (z) direction. The eigenvectors of the stiffness matrix are three perpendicular directions, along which the applied force induces a displacement aligned with the force. The eigenvalues K_1, K_2 and K_3 are denoted as the stiffness parameters of the fang structure.

Note that the explicit joint connection of the base of the fang to the chelicera and the muscles moving the fang introduce loads at their attachment areas result in complex boundary conditions. However, the use of a simplified fixed-base condition for the stiffness analysis is a valid approximation due to Saint-Venant's principle. Nevertheless, the approximated solution is limited in its ability to predict the actual stress distribution at the base. The effects of not perfectly-fix base conditions on the structural stiffness were also estimated by FE simulations. The results show only small changes in the absolute

stiffness of the structure, specifically ~1% for the conical- and ~10% for needle-like architecture.

Supplementary note 2. The spider fang built from composite material - elastic moduli of arrays with parallel-fibers and rotated-plywood fibers and the stiffness of the composite fang

We consider a fibril array composed of isotropic stiff fibrils (Young's modulus E_f, Poisson ratio ν_f, shear modulus $G_f = E_f/2(1+\nu_f)$ and a fibril volume fraction ϕ_f embedded in a more compliant isotropic matrix material (Young's modulus E_m, Poisson ratio ν_m and shear modulus $G_m = E_m/2(1+\nu_m)$). A range of matrix-to-fibril modulus ratios, $10^{-3} \leq E_m/E_f \leq 10^{-1}$, is considered for the fang material; a ratio of $E_m/E_f \approx 10^{-3}$ is typical for most of the fang length and $E_m/E_f \approx 10^{-1}$ represents the stiff region close to the tip where the matrix is rich in metal ions (2). The Poisson ratio for both materials is taken as $\nu_f = \nu_m = 0.3$. The exact fibril content in the spider fang is yet unknown and a range of $0.1 \leq \phi \leq 0.3$ considered (3).

The effective axial modulus and shear modulus of an array of parallel fibers (E_{Par} and G_{Par}) are evaluated here by the following classical Voigt and Reuss composite models (4):

$$E_{Par} = \phi E_f + (1-\phi)E_m \tag{3}$$

$$G_{Par} = \left[\frac{\phi}{G_f} + \frac{(1-\phi)}{G_m}\right]^{-1} \tag{4}$$

The effective axial and shear moduli of a rotated-plywood fibril array (E_{Ply} and G_{Ply}) are evaluated by an effective averaging over the moduli of the fibril sheets composing the array (4,5), as follows:

$$E_{Ply} = \frac{3}{8}E_{\parallel} + \frac{5}{8}E_{\perp} \tag{5}$$

$$G_{Ply} = \frac{1}{8}E_{\parallel} + \frac{1}{4}E_{\perp} \tag{6}$$

where $E_{\parallel}$ and $E_{\perp}$ are the axial moduli of an individual fibril sheet along and perpendicular to the fibrils, calculated by applying the Voigt and Reuss models :

$$E_{\parallel} = \phi E_f + (1-\phi)E_m \tag{7}$$

$$E_{\perp} = \left[\frac{\phi}{E_f} + \frac{(1-\phi)}{E_m}\right]^{-1} \tag{8}$$

Supplementary figure 2 plots the Young's modulus and shear modulus of parallel-fibered and rotated-plywood fiber arrays for a range of ratios for the matrix-to-fibril modulus, calculated by eqs. (3-8).

Note that for the case of $E_m/E_f \ll 1$eqs. (3-6) are reduces to:

$$E_{Par} \approx \phi_f E_f \tag{9}$$

$$G_{Par} \approx \frac{G_m}{(1-\phi)} \tag{10}$$

$$E_{Ply} = \frac{3}{8}\phi E_f \tag{11}$$

$$G_{Ply} = \frac{1}{8}\phi E_f \tag{12}$$

These asymptotic relations are obtained in supplementary figure 4 when $E_m/E_f \rightarrow 10^{-3}$. Eqs. (9-12) indicate that in case of $E_f \ll E_m$, the E_{Par}, E_{Par} and G_{Ply} moduli are dominated by the fiber stiffness but almost unaffected by variations in the E_m/E_f ratio. G_{Par} on the other hand is dominated by the matrix stiffness and monotonically decreases with E_m/E_f.

The stiffness parameters of the macroscopic layered fang structure are extracted using the classical laminate theory (4), by applying elastic moduli of the fiber arrays as evaluated above. Supplementary figure 3 demonstrates the variations in the penetration stiffness (K_1) and the perpendicular stiffness (K_3) of the fang-like model. The stiffness parameters are approximately proportional to the fibril content (ϕ) and only mildly affected by the stiffness ratio E_m/E_f.

Supplementary references

1. Timoshenko, S. *Strength of materials* (D. van Nostrand, New York, 1956).

2. Politi, Y., Priewasser, M., Pippel, E., Zaslansky, P., Hartmann, J., Siegel, S., Li, S., Barth, F. G. & Fratzl P. A spider's fang: How to design an injection needle using chitin-based composite material. *Adv. Funct. Mater.* **22** (12), 2519-2528 (2012).

3. Bereiter-Hahn, J., Matoltsy, A.G. & Richards, K. S. *Biology of the Integument. Volume 1. Invertebrates*, Chap. 32 ed. J. E. Hillerton, (Springer press, 1984).

4. Gibson, R. F. *Principles of composite material mechanics* (Mc-Graw-Hill, 1994).

5. Bar-On, B. & Wagner, H. D. Structural motifs and elastic properties of hierarchical biological tissues – A review. *J. Struct. Biol.* **183** (12), 149-164 (2013).
